# Integrated multimodel analysis of intestinal inflammation exposes key molecular features of preclinical and clinical IBD

**DOI:** 10.1136/gutjnl-2024-333729

**Published:** 2025-04-29

**Authors:** Miguel Gonzalez-Acera, Jay V Patankar, Lena Erkert, Roodline Cineus, Reyes Gamez-Belmonte, Tamara Leupold, Marvin Bubeck, Li-Li Bao, Martin Dinkel, Ru Wang, Laura Schickedanz, Heidi Limberger, Iris Stolzer, Katharina Gerlach, Leonard Diemand, Fabrizio Mascia, Pooja Gupta, Elisabeth Naschberger, Kristina Koop, Christina Plattner, Gregor Sturm, Benno Weigmann, Claudia Günther, Stefan Wirtz, Michael Stürzl, Kai Hildner, Anja A Kühl, Britta Siegmund, Andreas Gießl, Raja Atreya, TRR241 IBDome Consortium, Imke Atreya, Ahmed N Hegazy, Zlatko Trajanoski, Markus F Neurath, Christoph Becker

**Affiliations:** 1 Department of Medicine 1, Friedrich-Alexander-Universität Erlangen-Nürnberg, Erlangen, Germany; 2 Deutsche Zentrum Immuntherapie (DZI), Friedrich-Alexander-Universitat Erlangen-Nurnberg, Erlangen, Germany; 3 Department of Gastroenterology, Infectious Diseases and Rheumatology, Charité – Universitätsmedizin Berlin, corporate member of Freie Universität Berlin and Humboldt-Universität zu Berlin, Berlin, Germany; 4 Deutsches Rheuma-Forschungszentrum, ein Institut der Leibniz-Gemeinschaft, Berlin, Germany; 5 Department of Stem Cell Biology, University Hospital Erlangen, Friedrich-Alexander-Universität Erlangen-Nürnberg (FAU), Universitätsklinikum Erlangen, Erlangen, Bayern, Germany; 6 Department of Surgery, Universitätsklinikum, Friedrich-Alexander-University Erlangen-Nürnberg, Erlangen, Germany; 7 Biocenter, Institute of Bioinformatics, Medical University of Innsbruck, Innsbruck, Austria; 8 iPATH.Berlin, Charité – Universitätsmedizin Berlin, corporate member of Freie Universität Berlin and Humboldt-Universität zu Berlin, Berlin, Germany; 9 Department of Ophthalmology, Universitätsklinikum, Friedrich-Alexander-Universität Erlangen-Nürnberg, Erlangen, Germany

**Keywords:** INFLAMMATORY BOWEL DISEASE, IBD MODELS, CROHN'S DISEASE, ULCERATIVE COLITIS

## Abstract

**Background:**

IBD is a chronic inflammatory condition driven by complex genetic and immune interactions, yet preclinical models often fail to fully recapitulate all aspects of the human disease. A systematic comparison of commonly used IBD models is essential to identify conserved molecular mechanisms and improve translational relevance.

**Objective:**

We performed a multimodel transcriptomic analysis of 13 widely used IBD mouse models to uncover coregulatory gene networks conserved between preclinical colitis/ileitis and human IBD and to define model-specific and conserved cellular, subcellular and molecular signatures.

**Design:**

We employed comparative transcriptomic analyses with curated and a priori statistical correlative methods between mouse models versus IBD patient datasets at both bulk and single-cell levels.

**Results:**

We identify IBD-related pathways, ontologies and cellular compositions that are translatable between mouse models and patient cohorts. We further describe a conserved core inflammatory signature of IBD-associated genes governing T-cell homing, innate immunity and epithelial barrier that translates into the new mouse gut Molecular Inflammation Score (mMIS). Moreover, specific mouse IBD models have distinct signatures for B-cell, T-cell and enteric neurons. We discover that transcriptomic relatedness of models is a function of the mode of induction, not the canonical immunotype (Th1/Th2/Th17). Moreover, the model compendium database is made available as a web explorer (http://trr241.hosting.rrze.uni-erlangen.de/SEPIA/).

**Conclusion:**

This integrated multimodel approach provides a framework for systematically assessing the molecular landscape of intestinal inflammation. Our findings reveal conserved inflammatory circuits, refine model selection, offering a valuable resource for the IBD research community.

**WHAT IS ALREADY KNOWN ON THIS TOPIC**

- Preclinical modelling of IBD is key to the discovery of pathomechanisms and the evaluation of therapeutic approaches. However, individual models do not recapitulate the complexity of the disease, and comprehensive studies comparing modelling paradigms with human IBD are lacking.

**WHAT THIS STUDY ADDS**

- Our study provides a comparative analysis of 13 commonly used intestinal inflammation models, identifying core-conserved pathways between mouse models and IBD patient cohorts. Several key genes identified as IBD-associated through genome-wide association studies that regulate innate immune activation, cytokine signalling, neutrophil/monocyte recruitment, barrier integrity and metabolic homeostasis show regulatory conservation among the mouse models and patient datasets. In addition, our study shows novel model-specific cellular compositional changes in the transit amplifying, enteric nervous system (ENS) and B-cell compartments.

**HOW THIS STUDY MIGHT AFFECT RESEARCH, PRACTICE OR POLICY**

- By identifying conserved and discrepant pathways between specific mouse models and IBD patient cohorts, our analysis platform provides an invaluable resource for translational IBD research.

## Introduction

 IBD represents a group of debilitating and chronic gastrointestinal disorders that include Crohn’s disease (CD) and UC. Despite significant progress in our understanding of IBD, the precise aetiology remains elusive. IBD is multifactorial and features (a) an exaggerated and dysregulated immune response, (b) barrier dysfunction and microbial dysbiosis, (c) genetic susceptibility and (d) other unknown environmental causes.^1^,^3^ Recent genome-wide association studies (GWAS) have identified over 280 IBD-associated genomic loci, some that directly confer increased susceptibility to IBD.^4 5^ However, causal relationships have only been established for a few genetic variants, and the contributions of other factors in triggering IBD have suffered from the ‘chicken or the egg’ dilemma. This, together with the lack of shared molecular, prognostic and aetiological factors, has led investigators to hypothesise that IBD is a spectrum of diverse underlying disease mechanisms resulting in overlapping clinical presentation.^6^

To unravel the mechanisms underlying the complex nature of IBD, various mouse models have been established. Modelling complex diseases in preclinical models is challenging, yet enables a simulation of disparate disease aspects in a controlled context. Experimental models of intestinal inflammation are based on three broad approaches: (I) disruption of the intestinal epithelial barrier, (II) activation of an aberrant immune response and (III) alteration of microbial homeostasis. Each approach replicates specific components of disease aetiology, providing invaluable tools for exploring the IBD heterogeneity. However, none of these models fully reflect the complexity of human disease. This is exemplified by the distinct portrayal of particular pathobiological characteristics in each experimental model. Deciphering disease biology and developing novel therapeutic strategies therefore rely on the choice of an appropriate model to investigate the endophenotypes of concern.

Leveraging preclinical modelling of intestinal inflammation, here we generated transcriptomic data from 13 commonly used mouse models of intestinal inflammation to expose regulatory conservation in IBD-relevant molecular features and IBD-associated genes. By employing both correlative statistics and curation-based measures of comparison across the transcriptomes of each model, we uncovered shared and unique coregulated modules related to inflammatory response, carboxylic acid metabolism, mitochondriopathy and synaptic processes. Our findings offer a framework for comparing and selecting appropriate modelling paradigms that align with conserved disease processes, paving the way for discovering novel translatable therapeutic targets.

## Results

### Modelling transcriptomes across experimental intestinal inflammation

**Figure 1:**
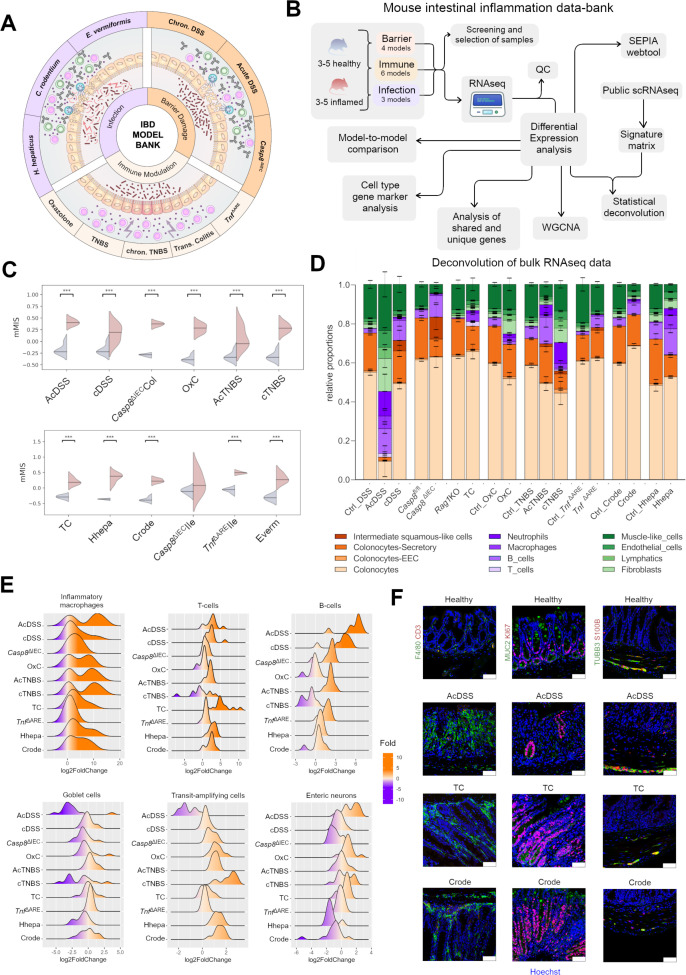
Changes in tissue cell composition across mouse models of IBD. (**A**) Scheme depicting the mouse model data bank for preclinical gut inflammation, highlighting the distribution of the broad categories: infection, barrier damage and immune modulation (**B**) Flowchart depicting establishment of the transcriptomic databank SEPIA for mouse IBD models including model screening selection, quality control (QC), and analyses steps. (**C**) Mouse mucosal Molecular Inflammation Score (mMIS) across models compared with respective controls (*** indicates padj <0.05, EB moderated t-test). (**D**) Deconvolved cell type signatures across colonic models. Inflamed samples are shown next to controls. The columns Ctrl_OxC and Ctrl_Crode are shared reference controls for the two respective models (**E**) Ridge plots of the fold change of selected cell type marker genes across the colonic mouse models. (**F**) Immunofluorescence staining for markers for macrophages (green, first column), goblet cells (green, second column), transit amplifying cells (red, second column), enteric neurons (green, third column) and enteric glia (red, third column) across the indicated models from each category. WGCNA, weighted correlation network analysis.

Modelling of intestinal inflammation in mice can be broadly classified into three categories: barrier damage, immune modulation and infection. To explore disease mechanisms, we recreated mouse models representing these categories on a C57BL/6J strain. We established cohorts for 13 models (figure 1A): AcDSS and cDSS colitis,^7 8^ *Casp8*^ΔIEC^Ile and *Casp8*^ΔIEC^Col ileitis and colitis^9 10^ for barrier damage; TC,^8 11^ OxC,^7 12^ AcTNBS and cTNBS,^7 13^ *Tnf*^ΔARE^Ile and *Tnf*^ΔARE^Col^14 15^ for immune modulation; Everm,^16 17^ Hhepa^18 19^ and Crode^12 20^ for infection. An overview of the models and abbreviation details is provided in the online supplemental table S1. Models were prescreened and selected for fully inflamed mice using colonoscopy, in vivo imaging, faecal bacterial load and posteuthanasia histology and transgene expression (onlinesupplemental figure S1AB and online supplemental table S1). Using tissues from inflamed mouse models and the respective uninflamed controls, we generated a bulk transcriptomic databank, analysed it via various statistical tools and created an exploratory web tool, denoted Shared Experimental Inflammation Data (SEPIA) (figure 1B). Apart from histological and preclinical scores (onlinesupplemental figure S1AB and online supplemental table S1), we established a mouse gut Molecular Inflammation Score (mMIS) adopting a strategy used recently for staging molecular inflammation in IBD and validated this on a publicly available cohort of DSS colitis time course of resolution^21^,^23^ (online supplemental figure S1C). All models, except *Casp8*^ΔIEC^Ile, showed significant elevation in mMIS (figure 1C).

Next, we used scRNA-Seq datasets^24 25^ and deconvolution algorithms^26^ to extract cellular compositional features from our bulk transcriptomes. In inflamed mice, higher immune cell proportions were evident, with distinct cellular compositions across models, including macrophage and neutrophil infiltration, colonocyte subtype loss and stromal population impacts concurrent to histological and endoscopic findings of epithelial erosion, hyperplasia and enhanced granularity (figure 1D, onlinesupplemental figure S1AB and online supplemental table S2).

New and unexpected insights included the detection of squamous-like epithelia in cDSS and *Casp8*^ΔIEC^Col, muscle-like cell reduction in *Casp8*^ΔIEC^Col, AcTNBS, Hhepa and Crode, and elevated B-cell signatures in AcDSS, cDSS and AcTNBS models (figure 1D and online supplemental table S2). A poor representation of certain cell types such as enteric neuroglia prompted us to take a curated approach for validating the deconvolution results. For this, we analysed marker gene expression representing specific cell populations (figure 1E,F and onlinesupplemental figure S2AC). These analyses confirmed the deconvolution data such as loss of secretory goblet cells in AcDSS, AcTNBS, Hhepa and Crode models and revealed new findings such as an elevation in the transit amplifying (TA) cell markers in the Hhepa, Crode and cTNBS colitis models, correlating with epithelial hyperplasia (figure 1E and onlinesupplemental figures S1B  S2A). Furthermore, we detected that enteric neuronal marker levels contrasted strongly between infectious versus the AcDSS and OxC models (figure 1E and online supplemental figure S2A). An interesting inverse correlation was detected between B-cell versus TA-cell markers, and a similar inverse trend emerged between enteric neuron versus TA-cell markers (figure 1E and online supplemental figure S2A). Corresponding trends at the protein level confirmed our transcriptomic findings (figure 1F).

In small intestinal models, we observed model-specific immune cell marker changes (online supplemental figure S2B). Strikingly, the *Tnf*^ΔARE^Ile model exhibited a strong inverse trend between T- and B-cell markers and was the only model with strong B-cell marker expression. Similar to colonic models, a negative correlation between the B-cell and TA-cell markers was also observed. Goblet cell markers were repressed in the Everm and *Casp8*^ΔIEC^Ile, matching histological findings (onlinesupplemental figures S1A  S2B,C). Interestingly, despite histological inflammation, the *Casp8*^ΔIEC^Ile model showed repression in T-cell signatures correlating with sterile inflammation owing to higher necroptosis-derived self-antigens in this model (onlinesupplemental figures S1A  S2B,C). Our analyses demonstrate mouse model-specific and shared cellular changes with novel negative correlations between TA cells versus B cells and enteric neurons.

### Common and unique transcriptomic signatures from diverse models simulate IBD pathways

**Figure 2:**
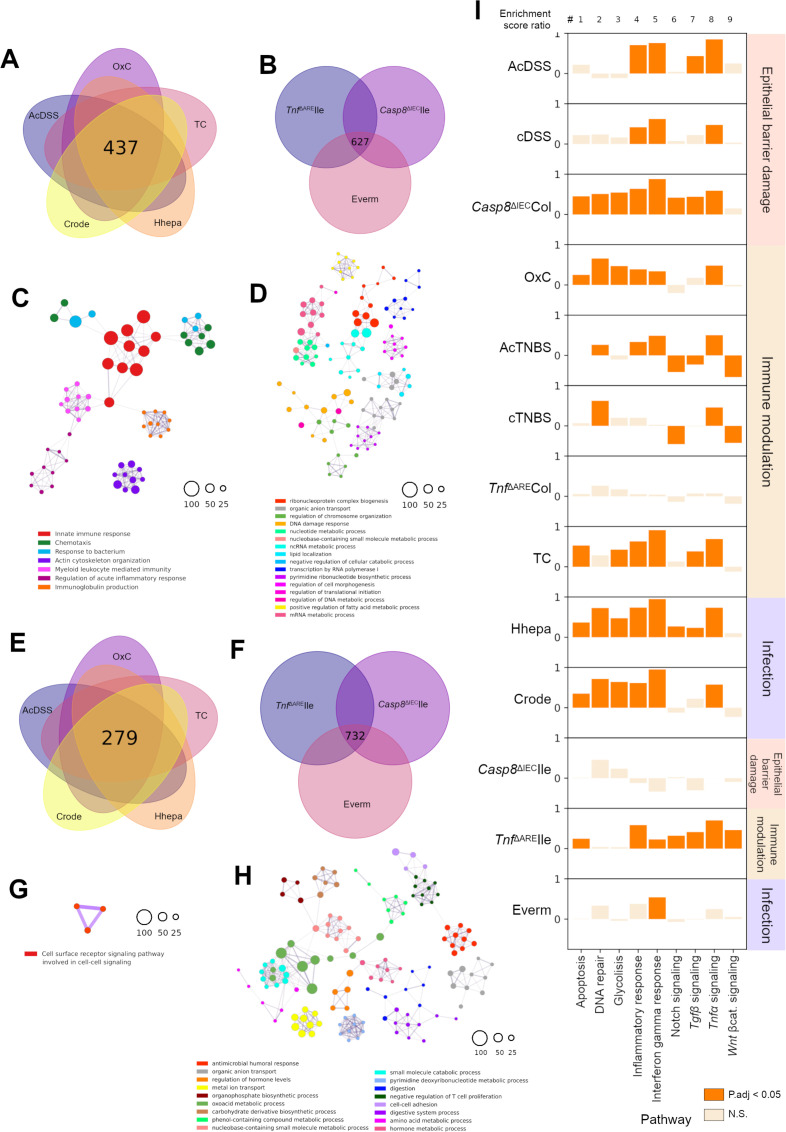
Regulatory commonalities and differences between mouse models. (**A**) Venn diagram showing the common upregulated genes between the indicated colitis models and (**B**) those between ileitis models. (**C–D**) Semantic similarity networks from the enriched GO terms observed in the upregulated gene set from the common datasets defined in A and B, respectively. (**E**) Venn diagrams showing the common downregulated genes between the indicated colitis models and (**F**) those between the ileitis models. (**G–H**) Term similarity network of the enriched GO terms observed in the downregulated gene set from the common datasets defined in E and F, respectively. (**I**) Variation in the relative expression of deconvolved hallmark pathways per mouse model showing variation profiles across the model dataset. GO, gene ontology.

To identify transcriptomic similarities between model categories, we performed correlative analyses yielding model relatedness distinct from the mode of inflammation induction (onlinesupplemental figure S3AC and online supplemental file 1). Next, we evaluated gene ontology (GO) similarities among coregulated genes in colonic (figure 2A, C=upregulated; 2E, G=downregulated) and small intestinal models (figure 2B, D=upregulated; 2F, H=downregulated). We analysed ontology-based semantic similarity to determine functional attributes between these genes.^27 28^ The 437 commonly upregulated genes in colonic models confirmed expected inflammatory responses, mainly including innate immune response, chemotaxis and response to bacteria (figure 2A,C). In contrast, the 627 commonly downregulated genes in small intestinal models showed upregulation in ribonucleoprotein complex biogenesis, mRNA metabolic process and DNA damage response (figure 2B,D).

The 279 commonly downregulated genes in colonic models (figure 2E) did not enrich against many ontology terms (figure 2G). In stark contrast, the 732 commonly downregulated genes in the ileal models (figure 2F) were enriched for antimicrobial humoral response, organic anion transport and regulation of hormone levels (figure 2H). Detailed ontology terms and adjusted p values are provided in online supplemental table S3. In addition to the shared genes and processes, we also analysed the unique genes specific to each model, identifying model-specific ontologies (onlinesupplemental figure S4AD and online supplemental table S3).

Next, we investigated the relationship between models and hallmark pathways associated with IBD using gene set variation analysis coupled with limma-based estimation of differences.^29^,^31^ Nine distinct hallmark pathways from the Molecular Signatures Database (MySigDB) relevant to inflammation and epithelial homeostasis were selected.^32 33^ The enrichment scores for specific pathways were highly similar for models within each category. In colonic barrier damage models, pathways like inflammatory response, interferon gamma (IFN-γ) response and tumour necrosis factor (TNF) signalling were robustly activated. Whereas in the colonic immune modulation category, DNA repair and TNF signalling pathways were activated (figure 2I). Crode and Hhepa infection models showed concordance in upregulated pathways, except for Wnt-β-catenin signalling, which was reduced in Crode but unaffected in Hhepa. Notch and transforming growth factor beta (TGF-β) signalling were upregulated in Hhepa but not in Crode (figure 2I). The AcTNBS and OxC models showed reduced Notch and Wnt-β-catenin signalling (figure 2I). It was striking that the TC model of immune modulation showed pathway enrichment similar to colonic infection models, with significant enrichment in most pathways, including TNF signalling (figure 2I). Among the small intestinal models, the TNF signalling reached significance only in the *Tnf*^ΔARE^Ile model, with a trend in the Everm, but not the *Casp8*^ΔIEC^Ile model (figure 2I). The IFN-γ response pathway was significantly enriched among all three small intestinal models (figure 2I). These analyses reveal the dominance of TNF and IFN-γ signalling across models and reveal the repression in Notch and Wnt signalling as unique to the AcTNBS and cTNBS colitis models.

### Gene coexpression modules in mouse models of intestinal inflammation

**Figure 3:**
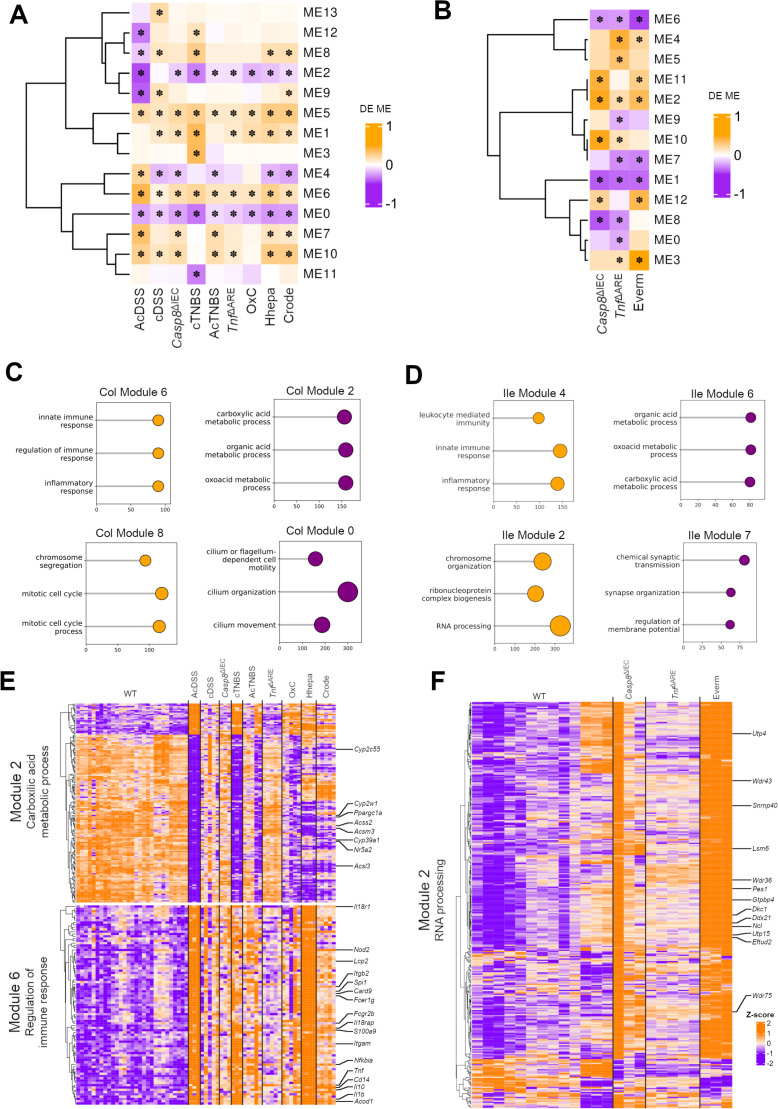
Weighted gene coexpression network analysis (WGCNA) across the mouse model datasets. (**A–B**) Variation observed in the modules obtained from the WGCNA applied to colitis and ileitis samples from the mouse model dataset. Changes labelled with ✽ indicate postlimma p values of <0.05. (**C–D**) Top three gene ontologies enriched in selected modules from the WGCNA of colitis (**C**) and ileitis, (**D**) respectively. (**E–F**) Heatmap of the enriched genes from the selected WGCNA modules from the colitis and ileitis mouse models, respectively.

To circumvent the limitations arising from pathway annotation and database selection, we employed weighted correlation network analysis (WGCNA)^34^ to identify gene coexpression modules across our mouse model datasets. The 14 colonic and 13 ileal modules of coexpression, thus identified (figure 3A,B and onlinesupplemental tables S4  S5), demonstrated the expected coexpression of specific modules. For instance, modules ME6 and ME4, respectively, were identified in both locations and consisted of GO biological processes related to ‘inflammatory responses’ (figure 3C,D). Interestingly, several of the co-upregulated genes in the colonic (figure 2A) and ileal (figure 2B) models fell into ME6 (colon) and ME4 (ileum) modules, respectively, consisting of immune regulatory ontologies with genes *Nod2*, *Nfkbia, Il1b*, *Il18r1*, *Lcp2* and *Itgb2*, among others (figure 3C–E and onlinesupplemental tables S4S6).

Furthermore, our analyses revealed that ME2 (colon) and ME6 (ileum) consisted of ontologies belonging to the carboxylic acid metabolic process, with notable regulatory genes associated with immune suppression (figure 3C–E and online supplemental table S6). For example, these modules included the gene *Nr5a2*, which encodes for LRH-1, a protein involved in the generation of immunomodulatory corticosteroids. They also included the genes *Cyp2w1*, *Cyp39a1* and *Cyp2c55,* which control cholesterol catabolism and supply for the synthesis of corticosteroids.^35^ It is well established that both host-derived and commensal carboxylic acid species regulate immune responses. Interestingly, the same modules also contained genes that regulate the supply of monocarboxylic acids, including, for example, *Acsm3*, *Acss2, Ppargc1a* and *Acsl3,* which are involved in the control of metabolic immunomodulation (figure 3C,E and onlinesupplemental tables S4  S6).^36^,^38^

In addition to the aforementioned common modules, tissue- and model-specific coregulated ontologies were also identified. For example, ontologies pertaining to cell projection morphogenesis were inversely correlated between AcDSS (up) and Hhepa (down) models (onlinesupplemental figure S4AB). Unique to the ileal models, ME2 consisted of upregulated ontologies for ‘RNA processing’ consisting of the genes *Wdr36*, *Dck1*, *Utp4* and *Wdr75* among others (figure 3D,F). These genes play a role in the context of p53 stress responses^39^,^42^ (figure 3D,F and onlinesupplemental tables S5  S6).

### Conserved and divergent coexpression ontologies and IBD-associated genes between preclinical models and IBD patients

**Figure 4:**
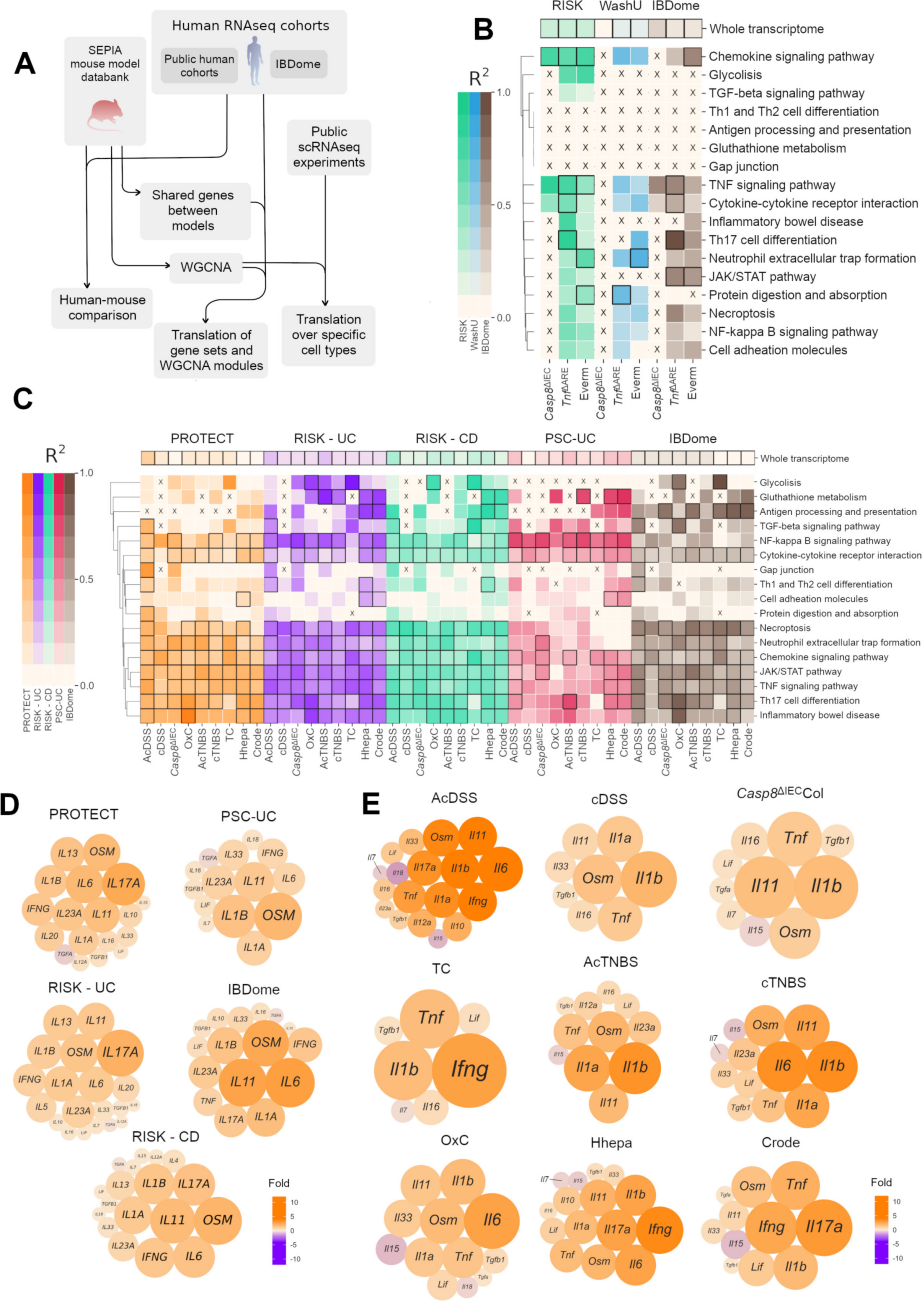
Regulatory conservation between mouse model and patient cohorts of IBD-associated features. (**A**) Flowchart depicting the analysis steps involved in statistical comparisons of transcriptomes generated from the SEPIA mouse model databank versus human IBD cohorts (**B–C**) Correlation sets for selected ontologies between (**B**) multiple mouse colitis models versus human UC cohort transcriptomes and (**C**) mouse ileitis models versus human CD cohort transcriptomes. Colour intensity represents the R^2^ value. Framed cells denote a significant p value in the regression test (p<0.05). Cells marked with an [X] had <10 genes in common, and the regression was not performed (**D–E**). Cytokine expression changes across (**D**) colonic human IBD cohorts and (**E**) colonic mouse inflammation models. Only cytokines reaching statistical significance (padj <0.05, Wald test) were included. The colour and the size of each bubble represent the expression fold change of each gene respective to control. The colour scale is constant between diagrams. The relative size scale represents absolute fold changes. CD, Crohn’s disease.

Comparative transcriptomics enables the identification of conserved disease mechanisms facilitating preclinical testing.^43^ We compared our mouse model data with public and own IBD-patient transcriptomic datasets (online supplemental table S7), analysing commonly regulated IBD-relevant ontologies^44 45^ (figure 4A). This revealed a number of interesting correlations between model–patient cohort pairs for specific IBD-relevant pathways from the Kyoto Encyclopedia of Genes and Genomes (KEGG), stratified by disease location: ileal (figure 4B) and colonic (figure 4C). The core pathways driving inflammation included chemokine signalling, cytokine receptor signalling, the Janus kinases/signal transducer and activator of transcription proteins (JAK/STAT) pathway and TNF signalling, with higher correlation across model–cohort pairs (figure 4B,C and online supplemental table S8).

Certain KEGG pathways showed specific correlations between model–cohort pairs. For instance, glutathione metabolism, cell adhesion and antigen processing and presentation exhibited a high R^2^ and met significance thresholds against infectious colitis models (figure 4C). However, among the small intestinal models, several orthologs did not reach the expected expression-enrichment thresholds (figure 4B, ‘x’ marks). A distinctive correlation pattern was observed for the pathway of gap junctions in the AcDSS model versus the PROTECT and IBDome patient cohorts. The Th1-Th2 differentiation pathway showed significant R^2^ for the Hhepa and AcDSS models versus most IBD cohorts (figure 4C and online supplemental table S8).

The Th17 differentiation pathway is an important player in the pathogenesis of IBD. This inflammatory pathway of Th17 cell differentiation showed an interesting trend, with significant to high R^2^ values. Among the colonic models, this pathway was significantly correlated in most of the model–cohort pairs, except the TC model (figure 4C). In the ileal models, Th17 cell differentiation was most prominent in the *Tnf*^ΔARE^ model (figure 4B).

The IBD cohorts in our study are broadly divided into paediatric (PROTECT and RISK)^46^,^51^ and adult (PSC-UC, WashU and IBDome).^52^,^55^ We identified specific correlations unique to paediatric cohorts, such as glycolysis in the *Tnf*^ΔARE^Ile and Everm models; TGF-β signalling in the TC and *Tnf*^ΔARE^Ile models; JAK/STAT signalling in the TC model and Th17 cell differentiation in the cDSS model (figure 4B,C).

Bulk transcriptomes enable the identification of tissue-wide cytokine landscapes. Cytokine expression levels were extracted from human and mouse model datasets for comparison, which yielded an overview of the most dominant cytokines by disease and modelling paradigm (figure 4D,E). The UC cohorts had a dominant expression profile for *OSM*, *IL17A, IL6* and *IL11* but showed *s*urprisingly little *TNF* expression (figure 4D). The CD cohorts also resembled this with the exception of the WashU cohort (online supplemental figure S5A). The mouse models showed distinct differences in cytokine profiles. Some expected changes in cytokine expression were the induction of *Ifng* and *Il17a* in the infectious models, the dominance of *Il33* in the *Casp8*^ΔIEC^Ile and the overall diversity in cytokines in the AcDSS model (figure 4E). Notably, specific colitis models that included cDSS, *Casp8*^ΔIEC^Col, AcTNBS, cTNBS and OxC, with dominant Oncostatin M *(Osm)* expression showed a lack of *Ifng* (figure 4E), which is in line with previous reports of OSM induction in neutrophils coupled with reduced IFN-γ secretion from T cells.^56^ Such an inverse correlation between *Osm* and *Ifng* was also observed in the ileitis models (online supplemental figure S5B). Overall, the data demonstrated the preponderance of *IL17A* and *OSM* in IBD datasets, whereas the mouse colitis models were marked by elevated *Il1b*.

Regulatory conservation between mouse models and human IBD extended to several mouse orthologues of the 283 IBD-associated genes identified in GWAS studies (IBD GWAS genes). Many of these genes were among the 437 commonly upregulated in all colonic models and mapped to modules ME6 and ME10 (onlinesupplemental figure S5C,D  S2A and online supplemental table S4). Although several genes were consistently downregulated across colonic models and exhibited regulatory overlap with the human UC dataset, PCK1 was the only IBD GWAS gene among them (onlinesupplemental figures S5E  S2E).

Expanding this analysis across mouse models and patient cohorts, we directly identified regulatory conservation of IBD GWAS genes. Most of these genes showed conserved upregulation in mouse models and patient datasets (online supplemental figure S5F). Key clusters emerged based on shared expression patterns across models, tissues and species. Notably, many of the conserved upregulated genes—such as *NOS2, LCN2, IL6, OSM* and *REG3A*—are well-established regulators of inflammation. A distinct cluster of IBD GWAS genes involved in epithelial transport and barrier function, including *ABCB1, SLC4A4, PCK1* and *AQP8*, exhibited conserved downregulation across models and species (online supplemental figure S5F).

Interestingly, UC-specific regulatory conservation also emerged, with genes like *PSTPIP1, RAC2, CD40, CIITA* and *CD28* showing consistent upregulation in both mouse models and UC patients (online supplemental figure S5F). In contrast, some of the IBD GWAS genes showed inverse regulatory patterns between mouse models versus patient cohorts, including *FADS2*, *MME*, *PPARG*, *RNF186* and *FFAR3*. These findings highlight both conserved and divergent gene regulation in key pathways, cytokines and IBD GWAS genes across mouse models and human IBD patient cohorts.

### Regulatory conservation of ontologies shows unique patterns of expression in IBD-associated cell clusters

**Figure 5:**
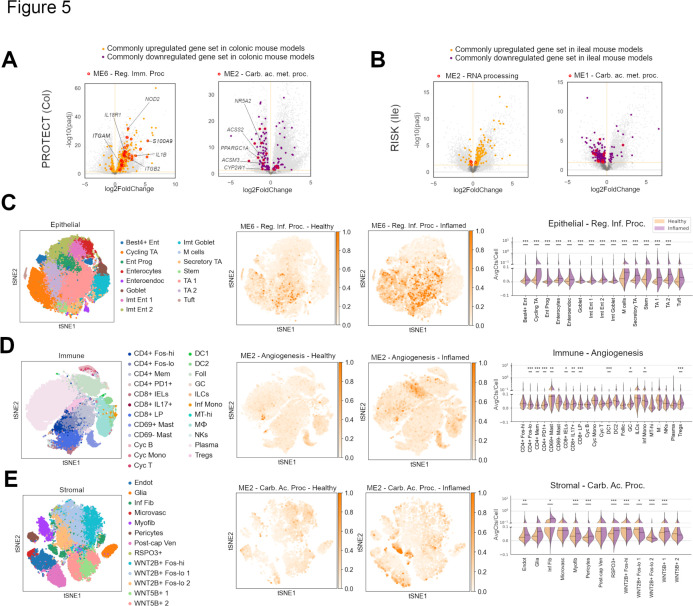
Conserved ontologies are expressed by unique IBD-associated single cell clusters. (**A**) Volcano plots of the indicated UC patient cohort where (left) orthologues from the common upregulated genes from colitis mouse models and (right) downregulated genes are shown. All genes commonly regulated among mouse colitis models and reaching the significance threshold of padj <0.05 (Wald test) are highlighted in orange or purple, and those belonging to the WGCNA mouse colitis modules ME116 and ME32 are labelled in red. (**B**) Volcano plots of the indicated CD patient cohort where (left) orthologues from the common upregulated gene set from ileitis mouse models and (right) downregulated genes reaching the significance threshold of padj <0.05 (Wald test) are highlighted in orange or purple. All genes commonly regulated among mouse models are highlighted, and those belonging to the WGCNA mouse ileitis modules ME2 and ME1 are labelled in red. (**C–E**) scRNA-Seq clusters of (**C**) epithelial, (**D**) immune and (**E**) stromal compartments from Smillie *et al*.^57^ Left: t-distributed stochastic neighbor embedding (t-SNE) clusters, middle: t-SNEs segregated by patient health where colour represents the average gene expression of selected pathways from the WGCNA modules, and right: violin plots showing the changes of the average expression in each of the annotated clusters. WGCNA, weighted correlation network analysis.

Among the genes which were commonly regulated between colonic models (figure 2A,E), several showed concordant regulation on the PROTECT (UC) patient transcriptomes (figure 5A). Interestingly, among these, several genes belonged to ME6 and ME2 (figure 5A, red highlights). Similarly, most genes commonly regulated among ileal models (figure 2B,F) also displayed regulatory conservation in the RISK (CD) cohort (figure 5B). However, the top ontology identified in the ileal WGNCA ME2, namely RNA processing, was not translated from mouse to patient dataset (figure 5B, red outlines). Among the top downregulated module ME1, we observed some regulatory conservation for the ontology ‘carboxylic acid metabolic process’ (figure 5B, red outlines). Thus, comparative transcriptomics between IBD patient samples and preclinical mouse models identifies regulatory conservation in pathways upregulating immune response and downregulating metabolism of carboxylic acids during inflammation.

Next, we investigated which cells from IBD patient tissues contribute most to these conserved ontologies affected by inflammation. For this, we leveraged the single-cell RNA sequencing (scRNA-Seq) dataset. Analysis of UC patient scRNA-Seq datasets^57 58^ revealed the emergence of cross-functional states of TA, stem and immature enterocytes which highly expressed genes from the colonic module ME6, with the GO ‘regulation of inflammatory process’ (figure 5C). In addition, microfold cells (M cells), which are critical for host defence, also showed an upregulation of this GO in the inflamed UC tissues (figure 5C). Another novel cellular state, specific to inflamed patients was a plasma cell subcluster. This subcluster displayed an elevated expression of genes enriched for the GO term ‘angiogenesis’ that was identified in the colonic module ME2 (figure 5D). Additionally, an elevated level of expression of the GO term ‘angiogenesis’ was observed on the CD69^+^ mast cells cluster specifically in inflamed UC tissues (figure 5D). To our surprise, a recently discovered novel stromal cluster of inflammatory fibroblasts and subclusters of endothelial and postcapillary venule endothelial cells exhibited higher average expression of genes belonging to the colonic module ME2 enriched for the GO ‘carboxylic acid metabolic process’ (figure 5E). We also performed an extended module-by-cell type analysis at the gene level for each colonic cell lineage (epithelial, immune and stromal) and stratified by disease status. We observed a notable degree of cell type specificity in the expression of genes from various modules such as ME9 (goblet), ME6 (cycling TA) and ME13 (M cells), in addition to other differences (online supplemental figure S6A). For example, expression of genes from ME13 with top enrichments related to squamous-like cells was highly enriched in inflamed UC M cells (online supplemental figure S6A and onlinesupplemental tables S6  S9). In addition, significant changes in specific modules were also observed in the stromal and immune compartments (onlinesupplemental figure S6BC and onlinesupplemental tables S6  S9).

The ileal module containing the GO ‘RNA processing’ failed to show any cell-cluster specific expression with comparable magnitude of expression across clusters from the scRNA-Seq data of CD ileal samples^59 60^ (online supplemental figure S7A). However, we detected other modules derived from ileitis mouse models that exhibited cellular-level alterations when compared with ileal CD scRNA-Seq clusters (online supplemental figure S7B and onlinesupplemental tables S6  S9). Collectively, these analyses highlight how conserved expression of specific ontologies is coupled to the emergence of specific cellular states during inflammation in IBD.

### Inflammatory impact on cellular substructures is conserved in specific model–cohort pairs

Aberrant transcript expression and physical organisation of intestinal epithelial cells substructures, such as microvilli, have been reported to affect patients with CD suggestive of epithelial dysfunction.^53^ Interestingly, analysis of our preclinical model data had also identified a common coexpression module, ME0, containing several ontologies related to ‘cilium’ (figure 3C). Interestingly, a parallel WGCNA module, ME1, was also identified in the adult colonic PSC-UC cohort that showed slight downregulation of ontologies related to ‘cilium’^52^ (onlinesupplemental figure S8AC). A module ME0 consisting of ontologies related to ‘cilium’ was also detected in the RISK ileum CD cohort but failed to reach statistical significance (online supplemental figure S8A). We recapitulated the original findings from the WashU ileum CD cohort where ciliary dysfunction was first reported, validating our analysis approach (onlinesupplemental figure S8AC). Confirming this, scanning electron micrographs of the epithelia from patients in our study cohort revealed microvilli damage and their disrupted organisation, supporting the finding that this feature is not only restricted to CD but can also be encountered in epithelia of UC patients (onlinesupplemental figure S8DE). However, it is noteworthy that none of the paediatric IBD cohorts, including RISK and PROTECT, had modules containing enrichments for ‘cilium’ or related ontologies. Moreover, cilium and related ontologies were significantly depleted in specific mouse models including the colitis models Crode, cTNBS and AcTNBS and all the ileitis models (onlinesupplemental figure S8FG). These transcriptomic findings correlated with physical damage to microvilli and their disrupted organisation observed in scanning electron micrographs in samples from the AcTNBS model (onlinesupplemental figure S8HI). Our data indicate that damage to epithelial microvilli is common in CD and UC, and this feature can be modelled in specific preclinical mouse models of intestinal inflammation.

Another subcellular organelle impacted in IBD is mitochondria. A recent study demonstrated how mitochondriopathy contributes to disease severity and treatment response in the UC PROTECT cohort.^47^ We replicated these findings in our in-house cohort where the expression levels of multiple mitochondrially encoded genes and the protein levels of COX4 were reduced in the UC patient samples (onlinesupplemental figure S8JK). Interestingly, extrapolation of these findings to our mouse model data showed that the mitochondrial changes were most closely paralleled in the infectious category, Everm and Hhepa, and to some extent in the *Tnf*^ΔARE^ Ile models (online supplemental figure S8L). It is noteworthy that in contrast to the infectious mouse models, the cTNBS colitis model showed an inverse correlation with respect to the expression of mitochondrially encoded genes.

Overall, our data highlight the conserved and divergent regulation of genes and pathways in preclinical intestinal inflammation models and clinical IBD cohorts. Using these as a tool for prescreening while targeting IBD processes will enable guided discovery efforts.

## Discussion

**Figure 6:**
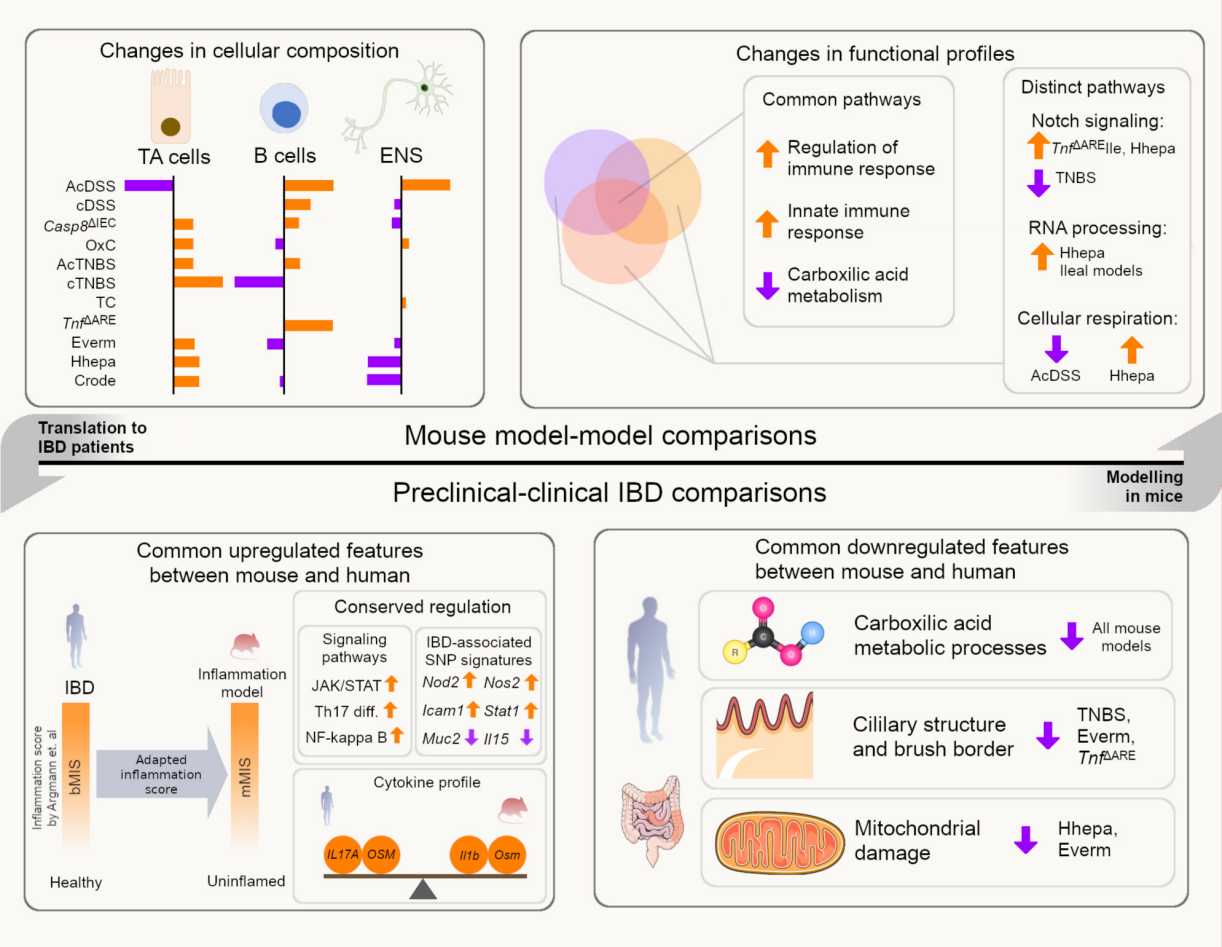
Schematic illustration of the key model-to-model and preclinical-vs-clinical IBD changes identified in the study.

Preclinical disease modelling to understand chronic inflammation is hindered by the poor translatability of results.^61^ A key challenge lies in selecting an appropriate model to investigate specific pathways or mechanisms. Given the diversity of models and different modes of inflammation induction, this choice becomes even more complex. This is particularly true in the case of researching IBD. Our comparative transcriptomic analyses highlight both shared and model-specific regulatory features, facilitating identification of translatable components between preclinical models and clinical disease (figure 6). Notably, we observed regulatory conservation in core inflammatory pathways, including JAK-STAT signalling, Th17 cell differentiation and NF-κB signalling, across mouse models and IBD patients. Many of these genes are part of the recently described biopsy molecular inflammation score (bMIS) for staging molecular inflammation in IBD.^21^ Leveraging this conservation, we adapted bMIS into the mMIS for preclinical models, which may enable a more standardised and unbiased evaluation of molecular mucosal inflammation.

Distinct cytokine landscapes define CD and UC. CD is driven by Th1/Th17 responses, with IFN-γ and IL-17/IL-22 as dominant cytokines, whereas UC exhibits a Th2-like profile with high IL-13 production.^62^ However, recent transcriptomic analyses challenge this classification.^63^ Our findings confirm the generalised elevation of TNF, IL-1β and IL-6 but identify OSM and IL-17A as the most dominant cytokines in IBD transcriptomes paralleled nicely on several mouse models. The discrepancy may emerge from previous studies reporting mucosal and circulating cytokine levels at the protein level, indicating that transcriptomes may not always reflect protein-level changes, with the need for further research to reconcile transcriptomic and proteomic findings. However, an integrative approach employing multiple mouse model- and human cohort-transcriptomes presents a robust framework for the identification of conserved and functionally relevant pathways contributing to inflammation.

In mouse models, cytokine expression showed both commonalities and differences. For example, *Il1b* was consistently upregulated, while infectious colitis models exhibited *Il17a* induction and shared antigen presentation features with human datasets. This reinforces *IL17A*’s conserved role in pathogen clearance and dysbiosis in IBD. Another notable finding was the repression of pathways regulating carboxy acid metabolites, a pattern conserved in human datasets. Though direct evidence linking these metabolites to inflammation homeostasis is limited, existing studies suggest an inverse relationship. For instance, corticosteroid-generating metabolites derive from these pathways,^35^ and downregulation of monocarboxylate transporter 1 impairs butyrate uptake, reducing colonocyte activation during intestinal inflammation.^64^

Although the overall immune regulatory alterations were easily traceable, environmental contributions to the model transcriptomes and their translation to patient datasets deem careful interpretation. For example, comparing mouse ileal models with human CD cohorts, we found that Everm and *Tnf*^ΔARE^Ile models recapitulated key immune activation pathways (Everm: TNF, IL-17, JAK/STAT; *Tnf*^ΔARE^: NF-κB, necroptosis). However, metabolic and epithelial barrier dysfunction were not recapitulated in these models. Overall, the *Tnf*^ΔARE^Ile model correlated the most with the human CD ileitis. Among colitis models, AcDSS and Hhepa infection correlated well with human UC datasets in inflammatory pathways. As in the ileum, glycolysis and glutathione metabolism remain generally poorly correlated across most model-to-cohort pairs, barring the infectious colitis models, suggesting a link between metabolic dysfunction and dysbiotic pathomicrobionts. Our findings, taken together with some recent studies, highlight the need for further research aimed at addressing the connection between glycolytic homeostasis and antimicrobial therapy in IBD.^65^ Additionally, the TC model, which correlated well with patient datasets in glycolysis regulation, aligns with emerging evidence on the role of glycolysis in mucosal T-cell immunometabolism.^66^ While these models capture key immune-driven aspects, additional or combinatorial approaches may be required to encompass the full IBD pathophysiology. Overall, integrating ileitis and colitis models with human cohorts confirms that no single model fully represents IBD, though *Tnf*^ΔARE^ (ileum) and DSS/TNBS (colon) best approximate their respective human diseases.

GWAS have identified ~280 IBD risk loci, primarily involved in immune regulation and epithelial homeostasis. A GWAS-guided strategy of creating genetic IBD mouse models yields translational insights into relevant disease mechanisms. Germline deficiencies in orthologues of IBD-associated genes (eg, *Il10*, *Muc2*, epithelial-specific *Atg16l1* deletion) recapitulate patient-like phenotypes. However, these models have a greater biological variation in the timing of inflammation onset, which is highly influenced by environmental and microbial influences and consequently were excluded in the current version of our model database.^67 68^ Moreover, a positive GWAS SNP association does not imply disruption of gene expression, and in some instances, a knockout in mice has yielded opposite phenotype.^69^ Conversely, genes like *TNF,* which lack IBD-associated SNPs, significantly impact pathology, disease course and therapy, emphasising the importance of gene regulation beyond GWAS associations. Owing to this, the constitutively active *Tnf*^ΔARE^ allele renders mice highly translatable for Crohn’s-like ileitis research and was thus included in our study.^70^ Research on regulatory conservation of IBD-associated genes in preclinical models remains limited. Our analysis reveals that several IBD GWAS orthologues that control innate immune activation, cytokine signalling, neutrophil and monocyte recruitment are conserved across models, independent of location or induction method, with significant overlap between species.

Diet-induced mouse models offer the potential to investigate the complex environment-gene interactions, which shape IBD pathogenesis. High-cholesterol diets trigger epithelial inflammasome activation,^71^ while high-fat/high-sugar diets, when combined with genetic or environmental factors, induce complex IBD pathomechanisms, including lipodystrophy, fat-gut crosstalk, oxidised fat-mediated cell death and immunometabolic dysregulation.^72^,^75^ However, full-blown inflammation in these models often requires secondary insults, limiting their use as primary IBD models. The complex role of the gut microbiota and microbial community structures greatly drives immune and inflammatory outcomes while modelling IBD. Thus, our study maintained specific pathogen-free conditions with regular screening to ensure cross-model comparability. Recent evidence suggests that conventional laboratory mice lack microbiota diversity relevant to inflammation research, whereas ‘wildling’ mice with natural microbiomes exhibit more representative immune responses. Despite their translational potential, wildling versions of common IBD models and genetic knockout strains remain unexplored, warranting future investigation.

By integrating coexpression module signatures with scRNA-Seq data from immune cell lineages in IBD patients, we identified a plasma cell cluster with lymphangiogenic functions, reinforcing recent work on angiogenic B-cell subsets by van de Veen *et al*.^76^ Additionally, inflammatory fibroblasts exhibited upregulation of carboxylic acid metabolic pathways, which modulate immune cell function.^77 78^ Notably, these pathways were repressed in our mouse model and IBD transcriptomes, aligning with emerging hypotheses that stromal cells in inflammation and cancer may produce immunosuppressive mediators.^79^,^81^ However, this could also reflect metabolic shifts driving extracellular matrix production. Further research is needed to clarify their role in paracrine immunosuppression during inflammation and remission.

Our study provides a resource and an interactive tool in the form of the SEPIA website for rapid transcriptomic stratification and selection of preclinical intestinal inflammation models, offering multiple analysis and data access options. These findings underscore key considerations for selecting appropriate mouse models to study disease-driving mechanisms and potential therapeutic targets in IBD. Moreover, our analyses highlight conserved and divergent regulation in key pathways and IBD GWAS genes, along with the interplay between the shared and model-specific inflammatory programmes, emphasising the need for further validation to define molecular IBD subgroups. A refined understanding of these regulatory similarities will enhance the translational value of preclinical models in studying intestinal inflammation.

## Materials and methods

Detailed materials and protocols are provided in the online supplemental methods section.

## Supplementary material

10.1136/gutjnl-2024-333729 online supplemental file 1

10.1136/gutjnl-2024-333729 online supplemental file 2

10.1136/gutjnl-2024-333729 online supplemental file 3

10.1136/gutjnl-2024-333729 online supplemental file 4

10.1136/gutjnl-2024-333729 online supplemental file 5

10.1136/gutjnl-2024-333729 online supplemental file 6

10.1136/gutjnl-2024-333729 online supplemental file 7

10.1136/gutjnl-2024-333729 online supplemental file 8

10.1136/gutjnl-2024-333729 online supplemental file 9

10.1136/gutjnl-2024-333729 online supplemental file 10

10.1136/gutjnl-2024-333729 online supplemental table 1

10.1136/gutjnl-2024-333729 online supplemental table 2

10.1136/gutjnl-2024-333729 online supplemental table 3

10.1136/gutjnl-2024-333729 online supplemental table 4

10.1136/gutjnl-2024-333729 online supplemental table 5

10.1136/gutjnl-2024-333729 online supplemental table 6

10.1136/gutjnl-2024-333729 online supplemental table 7

10.1136/gutjnl-2024-333729 online supplemental table 8

10.1136/gutjnl-2024-333729 online supplemental table 9

## Data Availability

Data are available in a public, open access repository. Data are available upon reasonable request.
