# Supplementary figures and images for: Integrated multimodel analysis of intestinal inflammation exposes key molecular features of preclinical and clinical IBD
Source: Gut. 2025 Apr 29;74(10):e333729. doi: 10.1136/gutjnl-2024-333729 (PMC12505074; doi:10.1136/gutjnl-2024-333729)

Supplementary figure 1

A

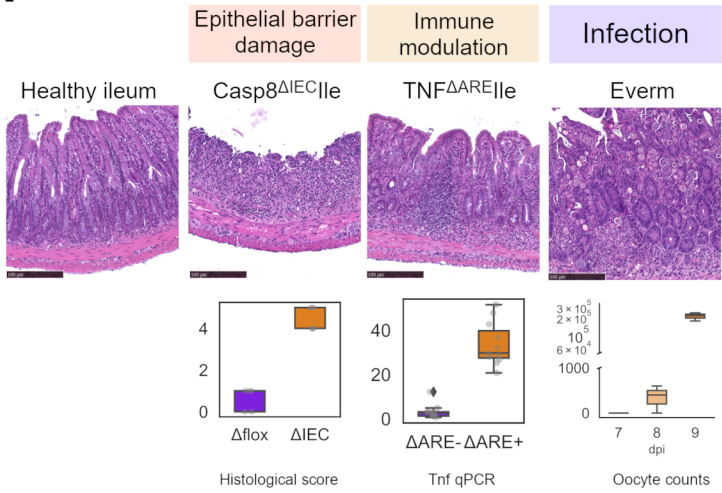

B

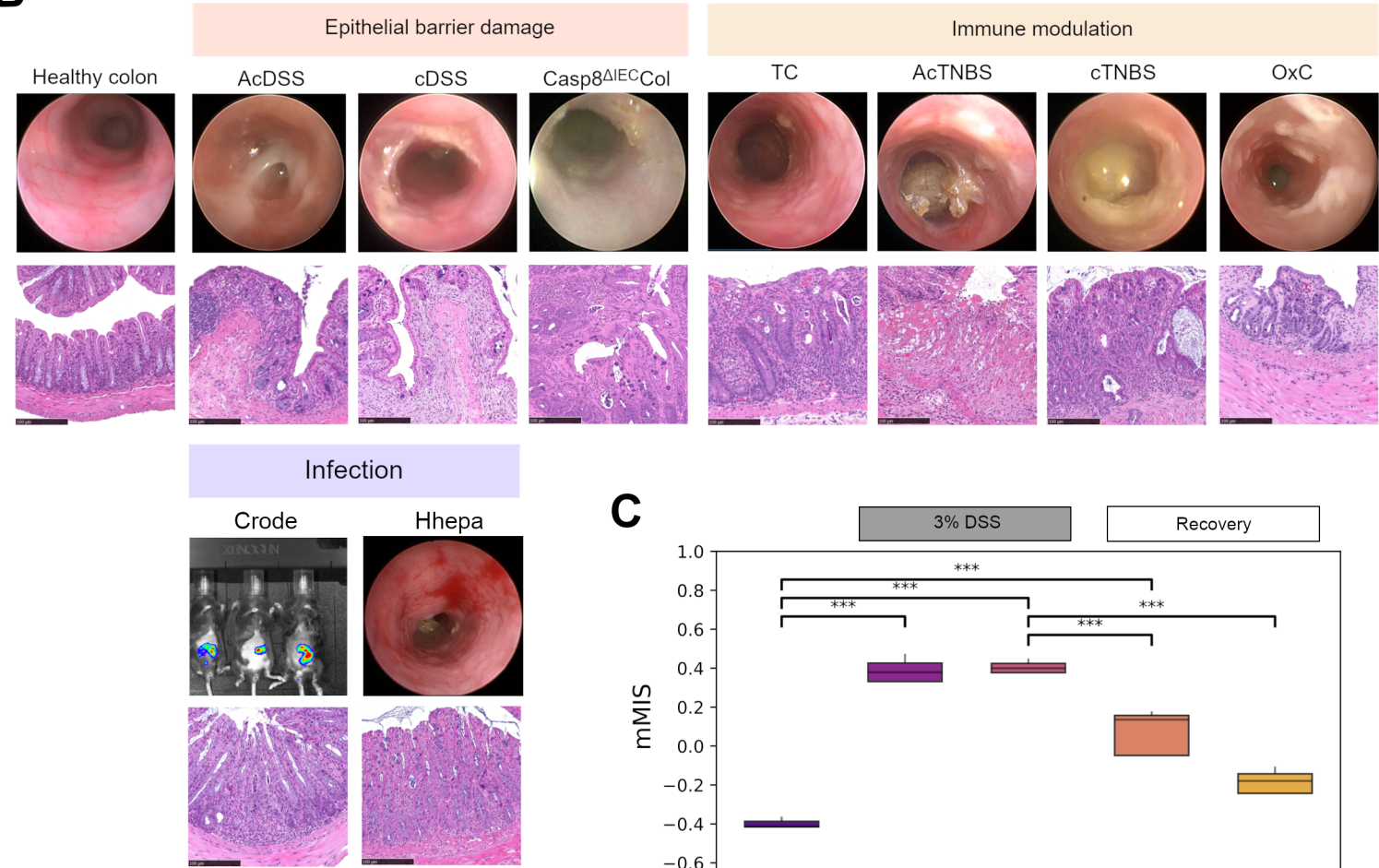

C

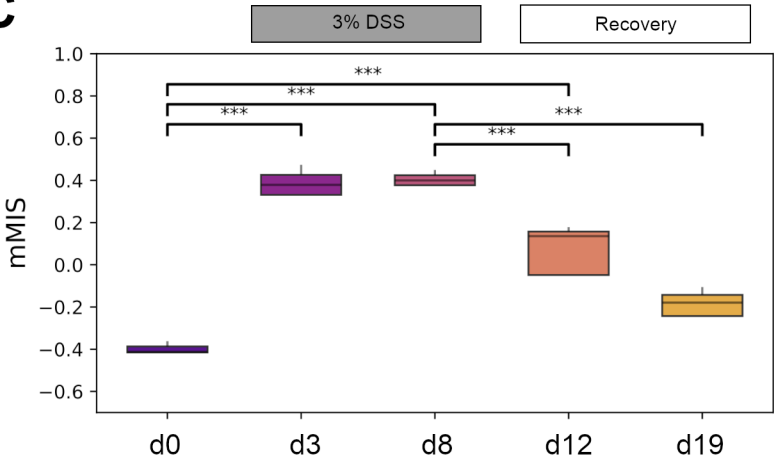

Supplement: online supplemental file 2 [file gutjnl-74-10-s002.pdf]

Supplementary figure 2

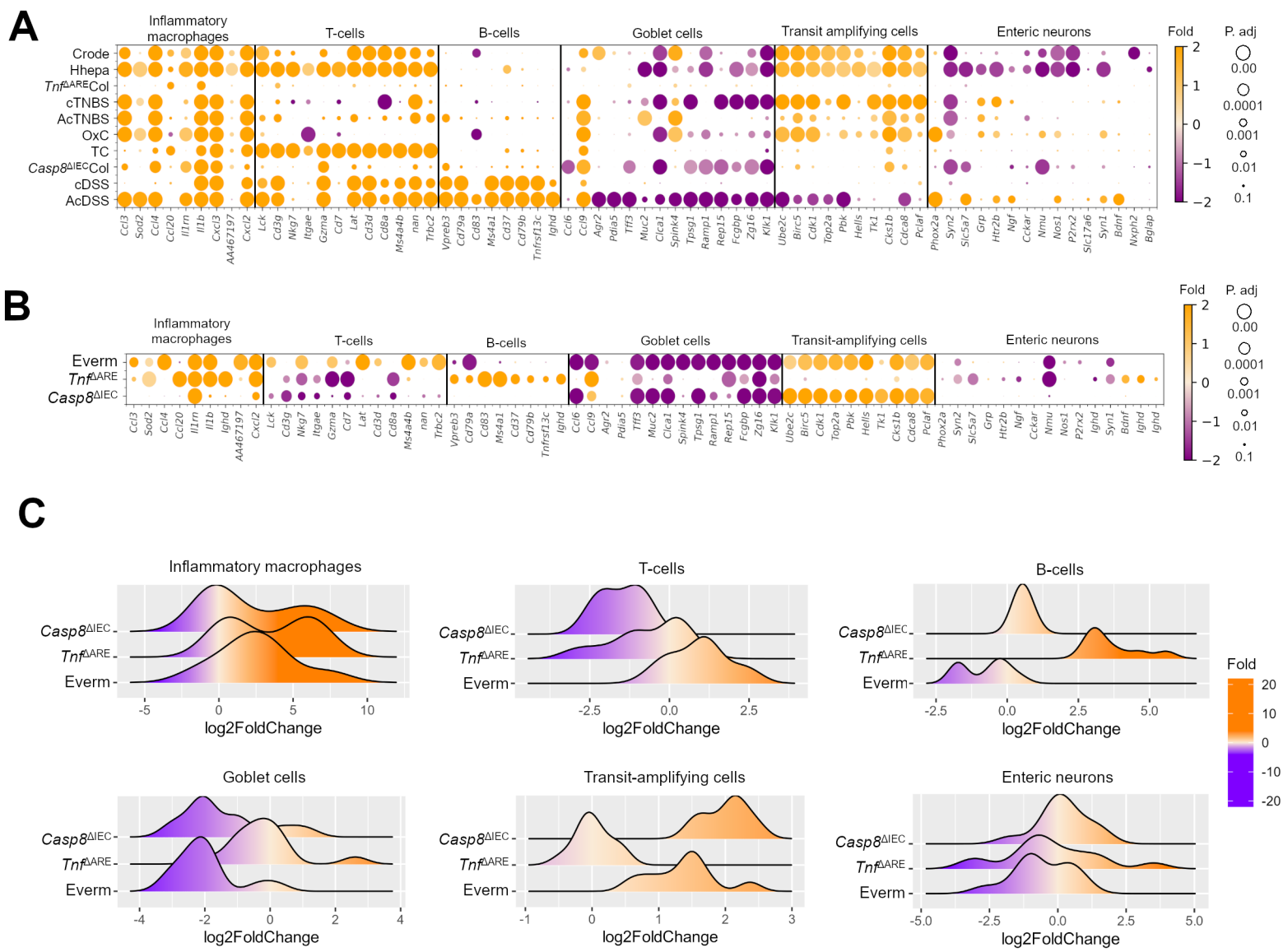

Supplement: online supplemental file 3 [file gutjnl-74-10-s003.pdf]

Supplementary figure 3

A

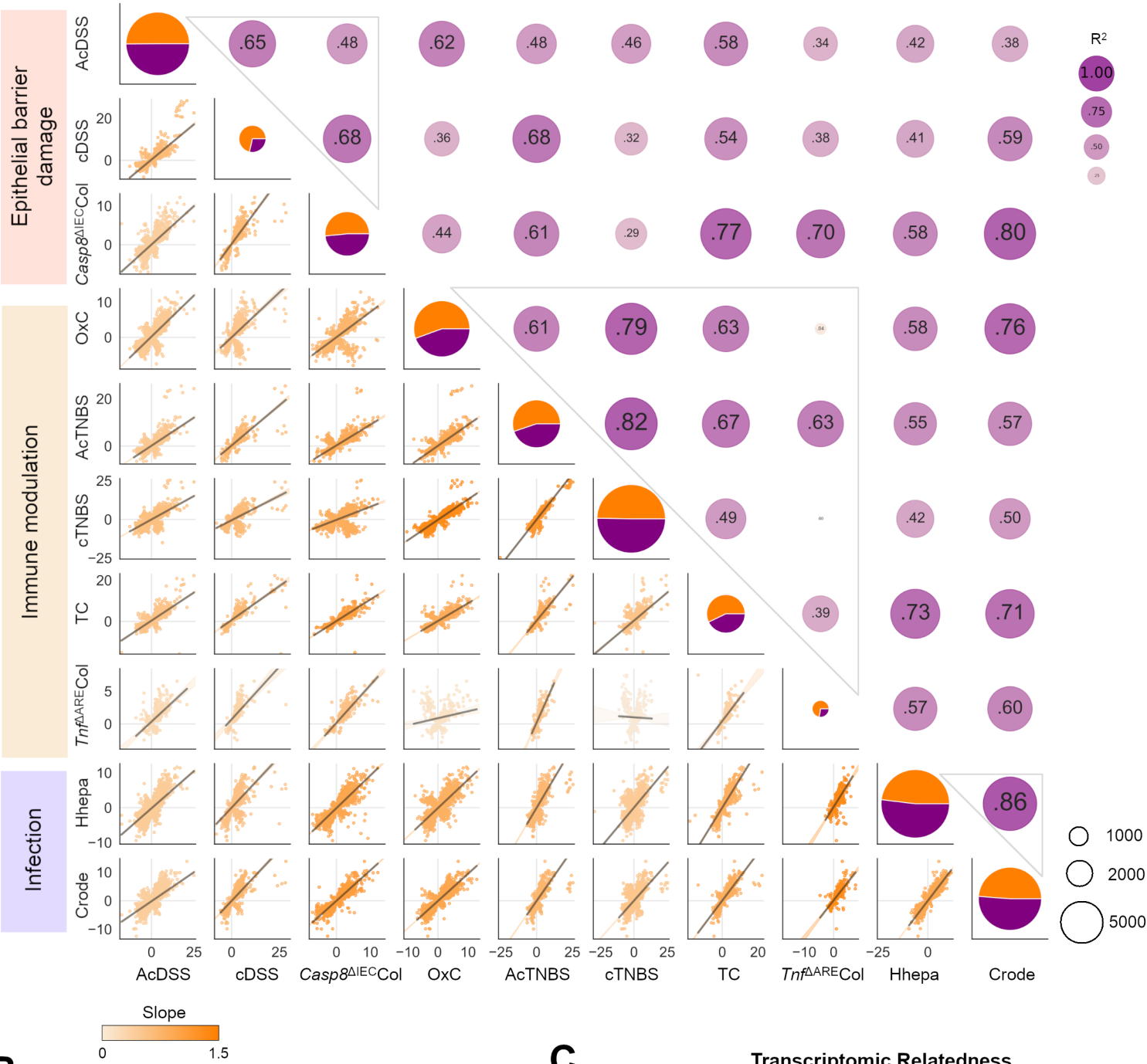

B

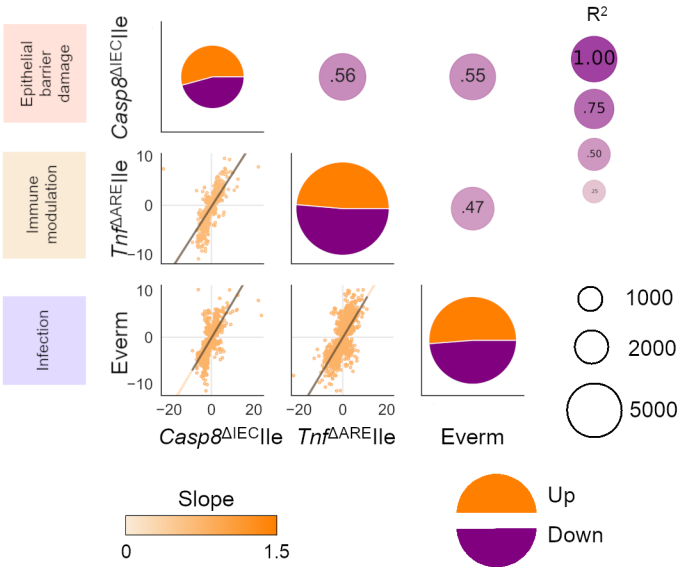

C

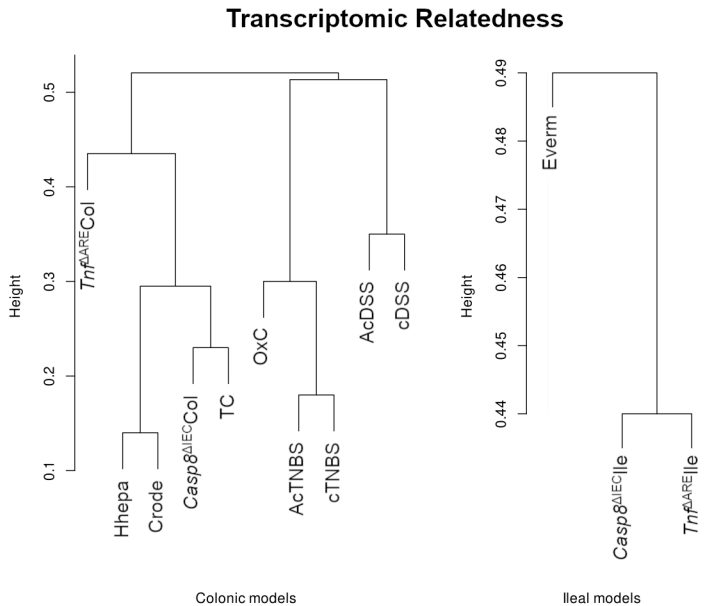

Supplement: online supplemental file 4 [file gutjnl-74-10-s004.pdf]

Supplementary figure 4

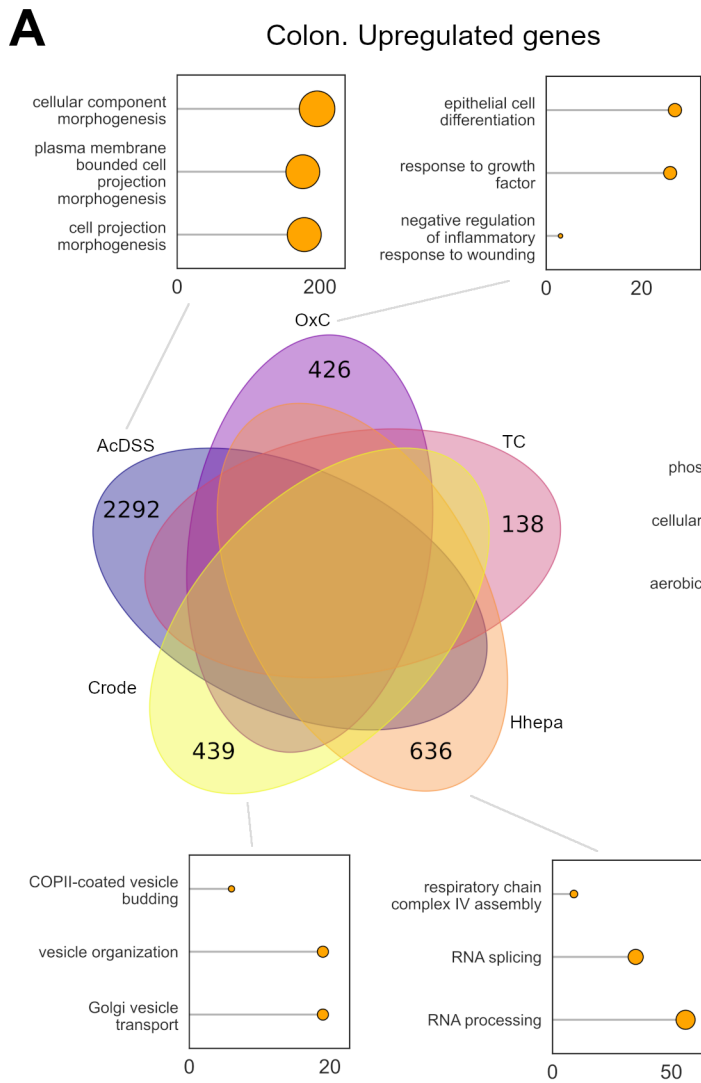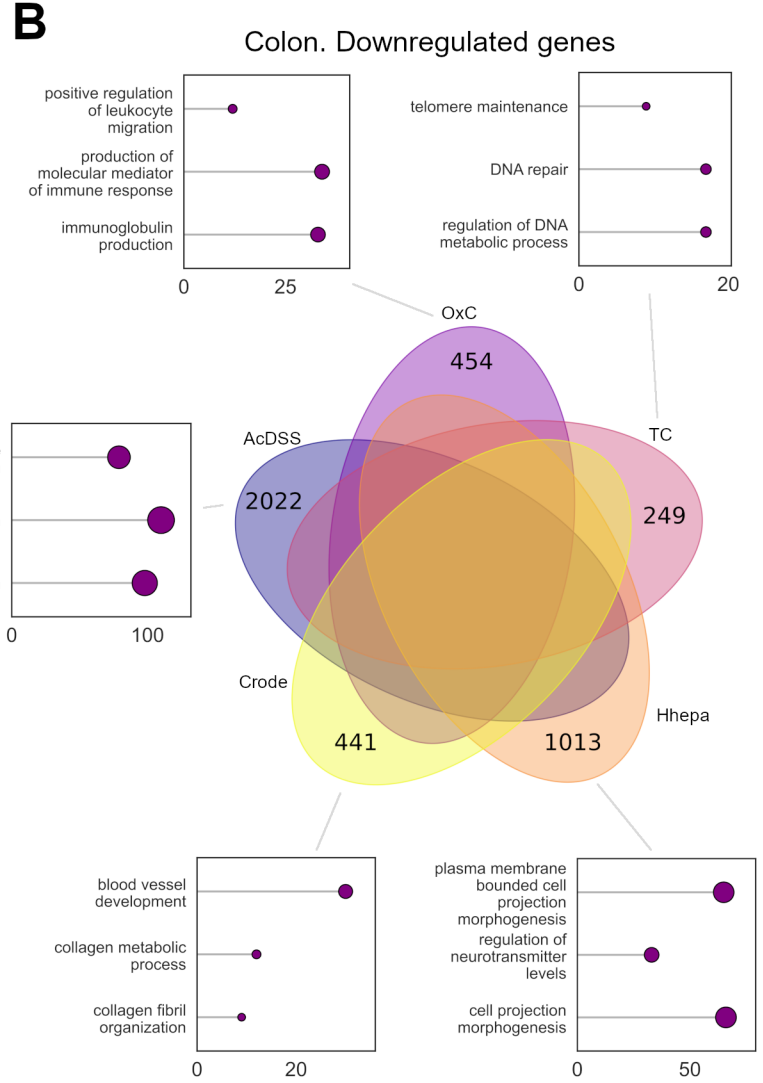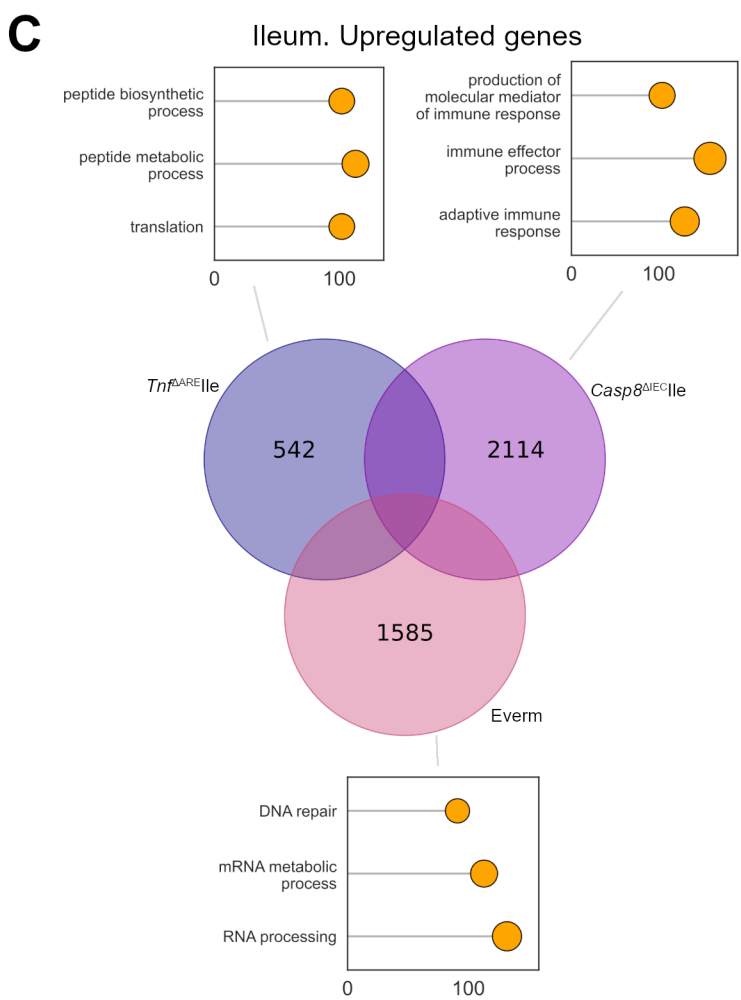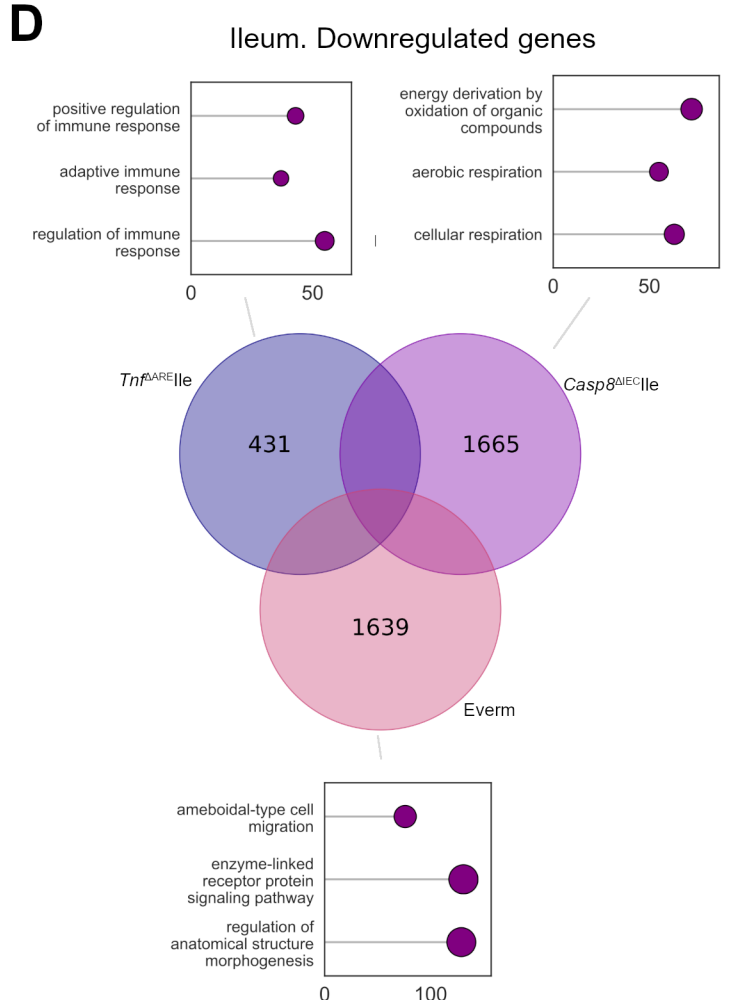

Supplement: online supplemental file 5 [file gutjnl-74-10-s005.pdf]

Supplementary figure 5

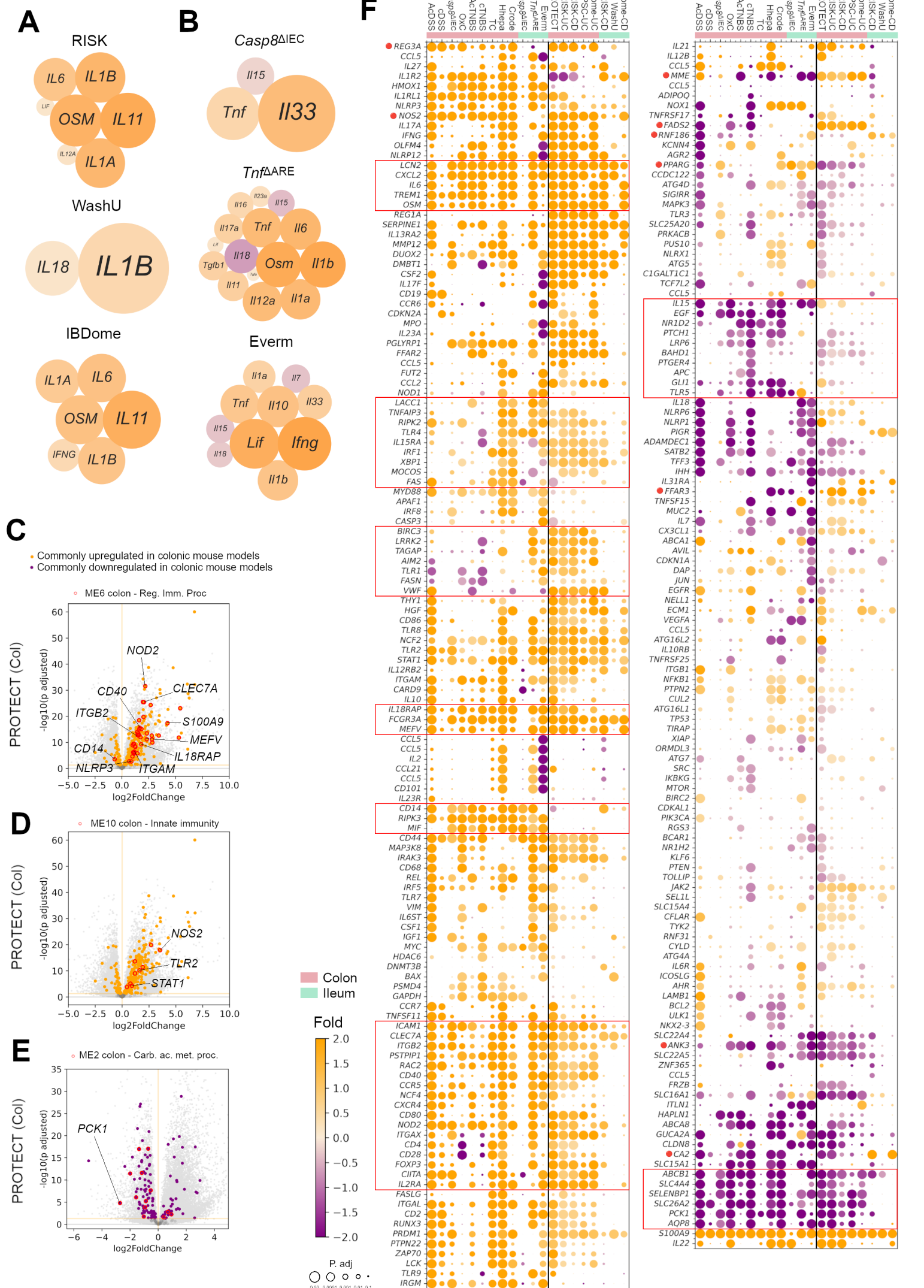

Supplement: online supplemental file 6 [file gutjnl-74-10-s006.pdf]

Supplementary figure 6

A

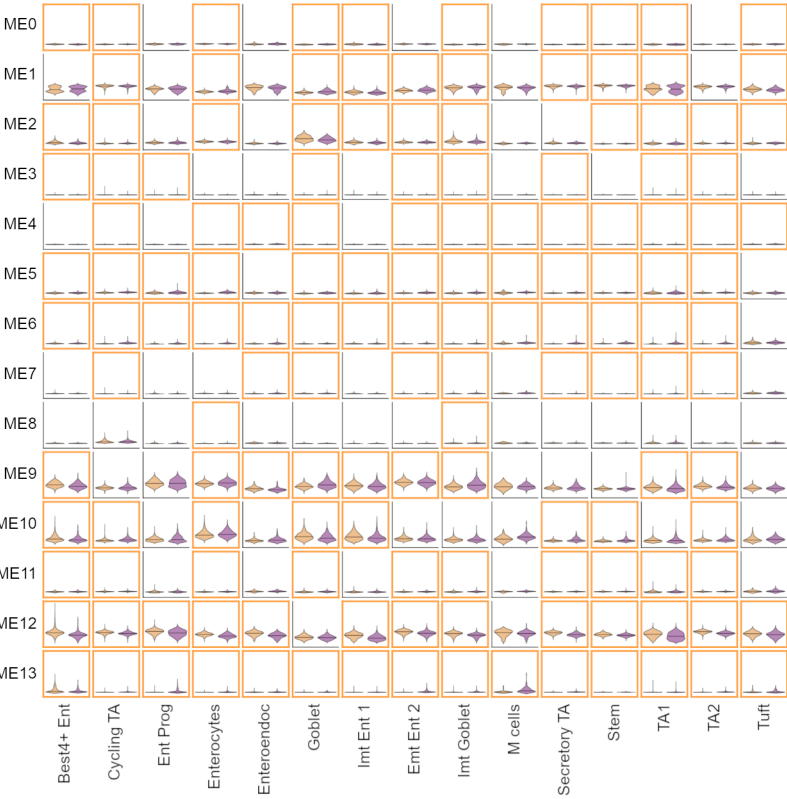

C

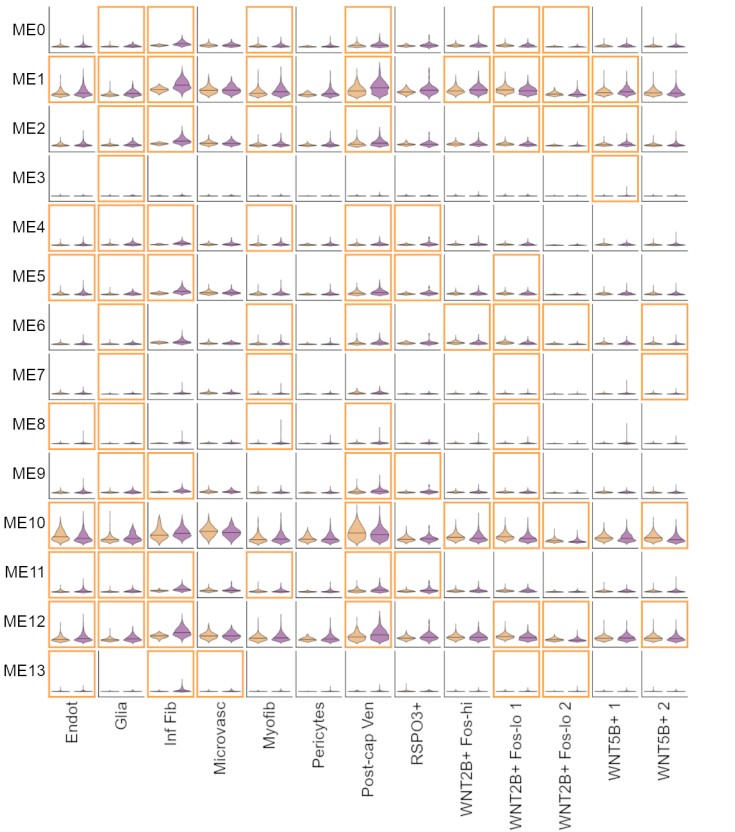

B

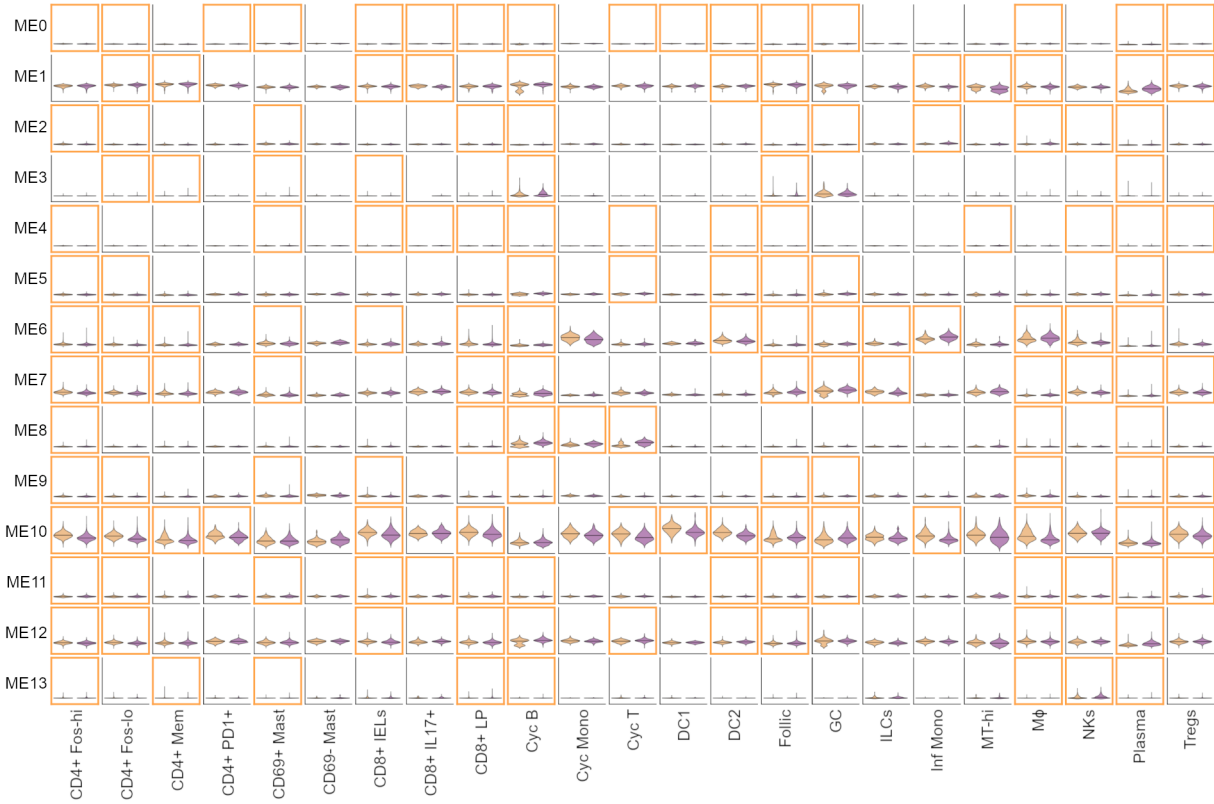

Supplement: online supplemental file 7 [file gutjnl-74-10-s007.pdf]

Supplementary figure 7

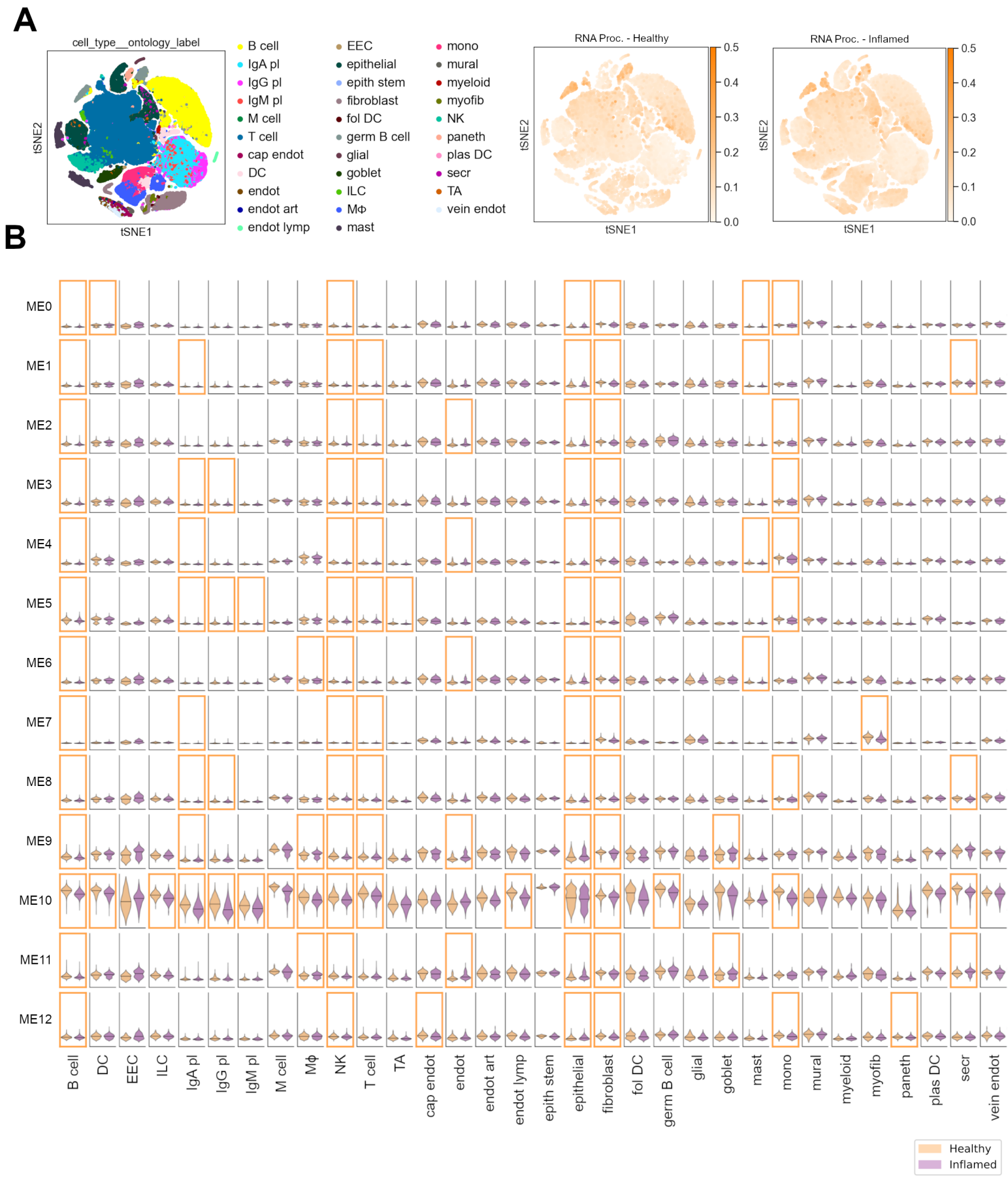

Supplement: online supplemental file 8 [file gutjnl-74-10-s008.pdf]

Supplementary Figure 8

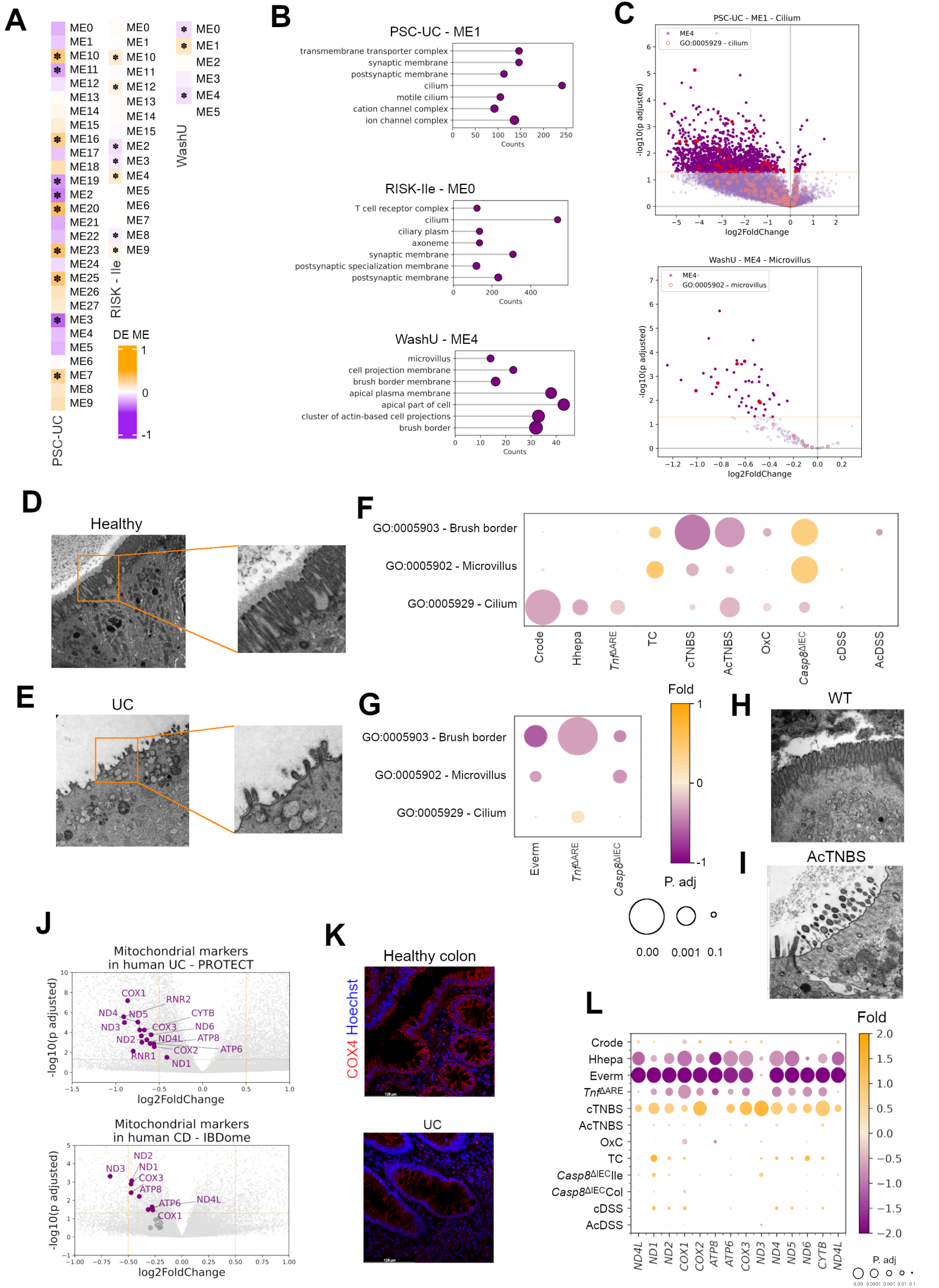

Supplement: online supplemental file 9 [file gutjnl-74-10-s009.pdf]
