# Supplementary material for: Integrated multimodel analysis of intestinal inflammation exposes key molecular features of preclinical and clinical IBD
Source: Gut. 2025 Apr 29;74(10):e333729. doi: 10.1136/gutjnl-2024-333729 (PMC12505074; doi:10.1136/gutjnl-2024-333729)
Supplement: online supplemental file 10 [file gutjnl-74-10-s010.pptx]

## Slide 1
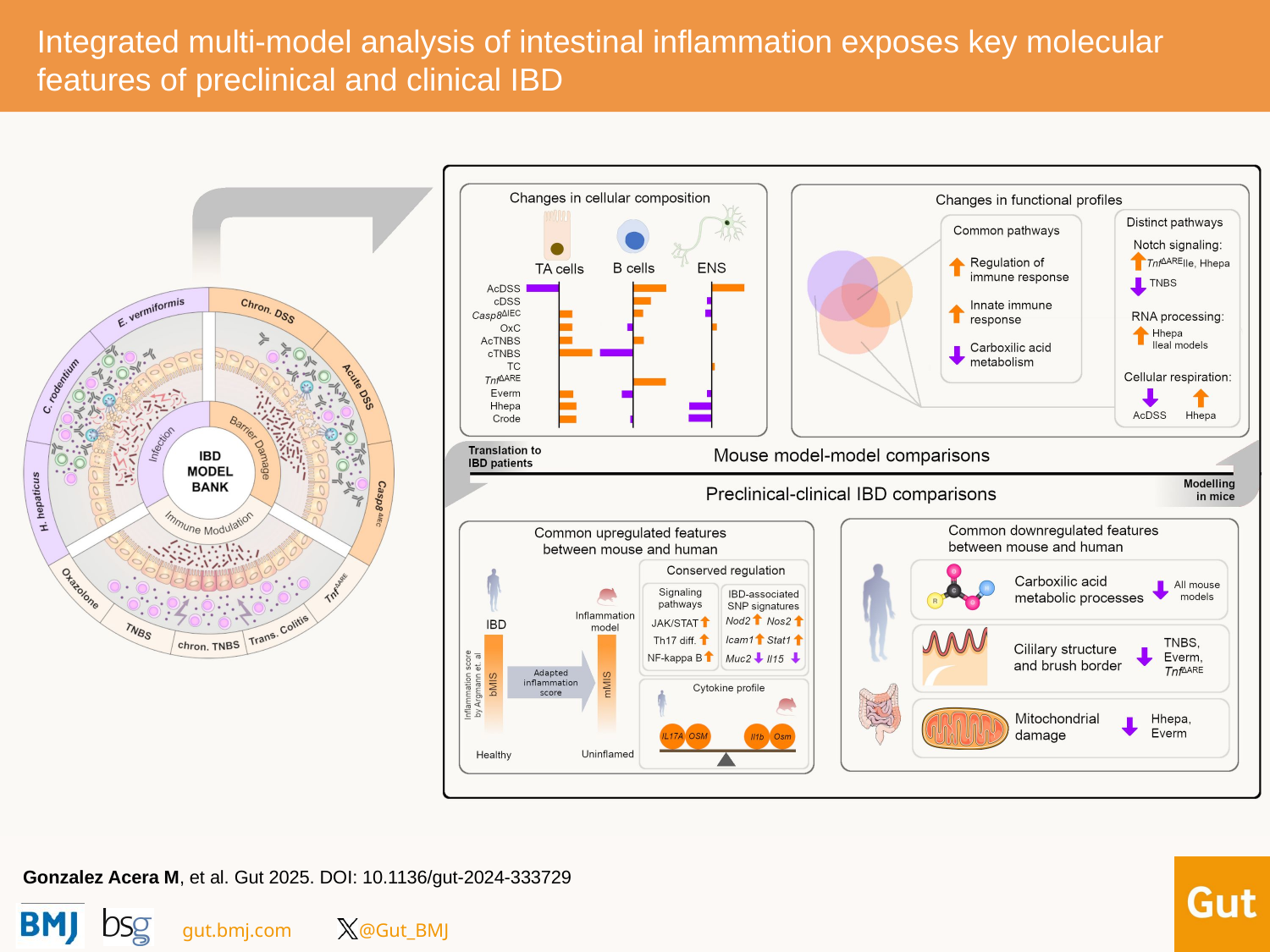

Integrated multi-model analysis of intestinal inflammation exposes key molecular features of preclinical and clinical IBD
Gonzalez Acera M, et al. Gut 2025. DOI: 10.1136/gut-2024-333729
gut.bmj.com
@Gut_BMJ
